# A chromosome-level genome assembly of the Asian house martin implies potential genes associated with the feathered-foot trait

**DOI:** 10.1093/g3journal/jkae077

**Published:** 2024-04-12

**Authors:** Yuan-Fu Chan, Chia-Wei Lu, Hao-Chih Kuo, Chih-Ming Hung

**Affiliations:** Biodiversity Research Center, Academia Sinica, Taipei 11529, Taiwan; Biodiversity Research Center, Academia Sinica, Taipei 11529, Taiwan; Biodiversity Research Center, Academia Sinica, Taipei 11529, Taiwan; Biodiversity Research Center, Academia Sinica, Taipei 11529, Taiwan

**Keywords:** feathered feet, ptilopody, Asian house martin, chromosome-level genome assembly, PacBio Hifi, Hi-C

## Abstract

The presence of feathers is a vital characteristic among birds, yet most modern birds had no feather on their feet. The discoveries of feathers on the hind limbs of basal birds and dinosaurs have sparked an interest in the evolutionary origin and genetic mechanism of feathered feet. However, the majority of studies investigating the genes associated with this trait focused on domestic populations. Understanding the genetic mechanism underpinned feathered-foot development in wild birds is still in its infancy. Here, we assembled a chromosome-level genome of the Asian house martin (*Delichon dasypus*) using the long-read High Fidelity sequencing approach to initiate the search for genes associated with its feathered feet. We employed the whole-genome alignment of *D. dasypus* with other swallow species to identify high-SNP regions and chromosomal inversions in the *D. dasypus* genome. After filtering out variations unrelated to *D. dasypus* evolution, we found six genes related to feather development near the high-SNP regions. We also detected three feather development genes in chromosomal inversions between the Asian house martin and the barn swallow genomes. We discussed their association with the wingless/integrated (WNT), bone morphogenetic protein, and fibroblast growth factor pathways and their potential roles in feathered-foot development. Future studies are encouraged to utilize the *D. dasypus* genome to explore the evolutionary process of the feathered-foot trait in avian species. This endeavor will shed light on the evolutionary path of feathers in birds.

## Introduction

Feathers are one of the most important and renowned traits serving varied functions across different regions of bird body. The majority of modern avian species have feathers covering most of their body, while their distal hindlimbs typically display scales. However, feathered feet, or ptilopody, sometimes manifest in avian species in which the ankle and foot are partially or completely covered with feathers (Bartels 2003). This trait is observed in certain birds, such as golden eagles (*Aquila chrysaetos*), northern hawk owls (*Surnia ulula*), and domesticated chicken breeds. While it was widely recognized as an uncommon trait in modern birds, the discoveries of feathers on the hindlimbs of basal birds *Sapeornis* and dinosaurs *Microraptor gui* have indicated that feathered feet could be a common trait in ancient birds (Xu *et al*. 2003; Zheng *et al*. 2013). This has fueled interest in the evolutionary origin and genetic mechanism underpinning foot feathering.

The study of feathered feet can be dated back to 1928 when Dunn conducted a review on the crosses of chicken breeds, revealing that feathered feet were determined by mutations at specific loci following Mendelian inheritance (Dunn 1928). Later, several studies have attempted to identify potential genes and pathways associated with this trait (Dhouailly *et al*. 1980; Widelitz *et al*. 2000; Domyan *et al*. 2016; Lai *et al*. 2018; Boer *et al*. 2019; Bortoluzzi *et al*. 2020; Li *et al*. 2020; Cooper and Milinkovitch 2023). Many of them have suggested that feathered feet in domestic birds were stemmed from expression changes of genes, PITX1 and TBX5, which are related to hindlimb and forelimb identity during embryonic development, respectively (Domyan *et al*. 2016; Boer *et al*. 2019; Bortoluzzi *et al*. 2020; Li *et al*. 2020). These gene expression changes result in the shift of hindlimb to forelimb-like identity (Domyan *et al*. 2016; Boer *et al*. 2019; Saxena and Cooper 2021). Alternatively, others found that changes in the WNT/β-catenin and retinoic acid pathways can modulate the conversion of scales to feathers in chicken feet (Widelitz *et al*. 2000; Lai *et al*. 2018). These findings contribute to our understanding of the mechanisms underlying feathered feet development in domestic birds.

However, the majority of research on feathered feet has focused on domestic species (e.g. Domyan and Shapiro 2017; Boer *et al*. 2019; Bortoluzzi *et al*. 2020; Li *et al*. 2020). These domestic populations might experience artificial selection and some of their feathered feet were intentionally selected by breeders (Bartels 2003). Consequently, the genetic mechanisms indicated by these studies might not fully explain the evolution of feathered feet in natural avian species. Therefore, to gain insight into the evolution of feathered feet in birds, there is a need to explore the genetic mechanisms of feathered feet in natural populations.

**Fig. 1.**
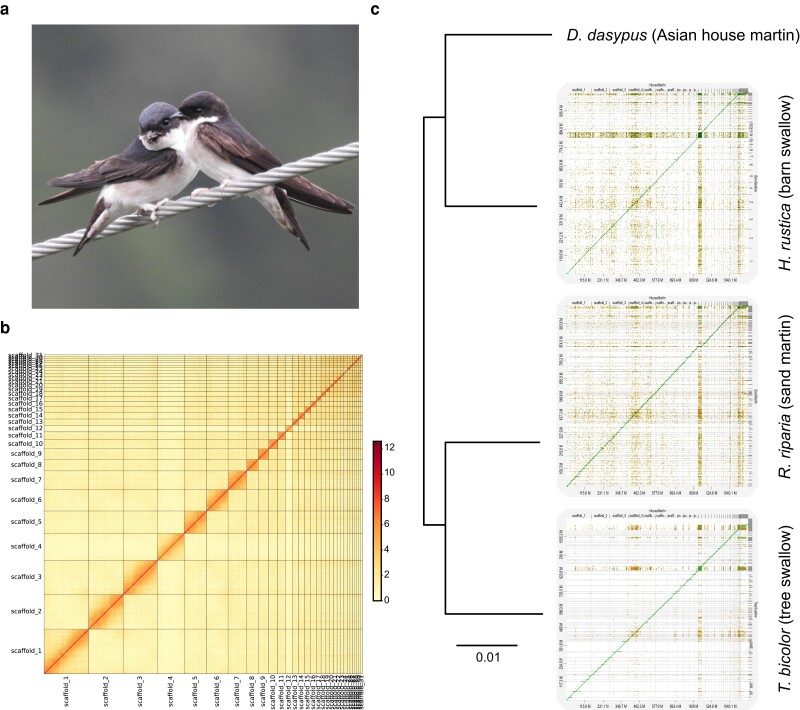
a) The Asian house martin (*D. dasypus*). b) The Hi-C heatmap of the *D. dasypus* assembly displays 31 chromosome-level scaffolds. The gradient of darkness indicates the contact frequency of two points, with darker indicating higher frequencies of contacts. c) The phylogenetic relationship and the sequence divergence rate of four swallow species inferred from minimap2. The diagrams display the D-genies dot plot of whole-genome alignment of each swallow species against *D. dasypus*. The *x*-axis indicates the chromosomes of the Asian house martin genome, while the *y*-axis indicates the scaffolds of the other swallow genome assemblies. The line along the diagonal suggests high synteny between house martin scaffolds and barn swallow chromosomes with high sequence identity (>75%). Photo credit: Chih-Ming Hung.

The Asian house martin (*Delichon dasypus*), with its genus distinguished from all other swallow genera by having white-feathered feet (Billerman 2022; Fig. 1a and Supplementary Fig. 1), presents an ideal candidate to investigate the origin and evolution of foot feathering in wild avian species. Here, we assembled a chromosome-level genome of the Asian house martin using PacBio HiFi reads combined with Hi-C technologies. We conducted a whole-genomic alignment of *D. dasypus* with other swallow species that have scale-covered feet, aiming to explore potential genes linked to the formation of foot feathering. We hypothesize that genomic regions exhibiting high sequence or structural divergence between *D. dasypus* and each of the other swallow species harbor genes or regulatory elements associated with the development of feathered feet.

## Materials and methods

### Sample collection

We collected Asian house martins at Lishan, a montane region in Taiwan (24°19′36.2″ N, 121°18′24.6″ E; *ca*. 1,900 m) using mist nets. For this study, we selected a female bird for de novo assembly, aiming to obtain both Z and W chromosomes. We collected blood samples from this bird, and extracted whole-genomic DNA using the Qiagen Purgene Cell kit. Additionally, pectoral muscle samples from the same bird were collected and immediately preserved in liquid nitrogen to investigate the 3D conformation of chromatin. Moreover, we collected eight different tissue types (muscle, heart, liver, kidney, lung, ovary, blood, and brain) from the same individual and extracted total RNA from each tissue using the Qiagen RNeasy fibrous tissue mini kit. The ethical approval was given by the Institutional Animal Care and Use Committee of Academia Sinica (Prove ID: 19-12-1402). The sample collection was approved by Taichung City (Permit no. 1090325836) and Tri-mountain National Scenic Area (Permit no. 1090002892). The specimen was vouchered in Biodiversity Research Museum, Academia Sinica (Voucher code: ASIZA0002281).

### Whole-genome sequencing

We sent both the genomic DNA and frozen pectoral muscle samples to the NGS High Throughput Genomic Core at the Biodiversity Research Center, Academia Sinica (Taipei, Taiwan), for sequencing. DNA libraries were prepared for circular consensus long-read sequencing using the High Fidelity (HiFi) SMRTbell library preparation method (Wenger *et al*. 2019). The long-read libraries were sequenced with two 1 M Single-molecule real-time (SMRT) cells on the PacBio Sequel platform and one 8 M SMRT cell on the PacBio Sequel IIe platform.

To investigate 3D chromatin conformation, we used the Phase Genomics Proximo Hi-C V4 kit for library construction and sequenced the sample on the Illumina HiSeq 2500 platform to obtain 2× 150 paired-end reads.

The RNA samples from eight tissue types were pooled, ensuring even concentration, and were sent to GENOMICS (Taipei, Taiwan) for RNA sequencing (RNA-seq). We used the Truseq Stranded mRNA kit for library preparation and conducted RNA-seq on the NovaSeq 6000 platform for 2× 150 paired-end reads.

### De novo genome assembly

Inputted with all HiFi reads (quality > Q20) generated from the three PacBio SMRT cells, we performed primary contig assembly using the default setting of Hifiasm version 0.19.6-r595 (Cheng *et al*. 2021). We removed allelic variants of two chromosomes that were falsely assembled as duplicate contigs using the stand-alone purge_dups program (Guan *et al*. 2020) with parameters −l5, −m20 and −u72. We then employed the Hi-C-informed scaffolder yaHS version 1.2 (Zhou *et al*. 2023) to achieve chromosome-level genome assembly. Prior to scaffolding, Hi-C reads were processed with HiC-Pro (Servant *et al*. 2015) to filter out read pairs that had low mapping qualities (MAPQ <10), represented dangling-ends or self-circles, or arose as polymerase chain reaction (PCR) duplicates.

### Assembled genome quality

To assess the quality of the assembled genome, we employed Quast version 5.0.1 (Gurevich *et al*. 2013) to calculate key assembly metrics, including the assembly size, scaffold number, and scaffold N50. We also used BUSCO version 5.2.2 (Manni *et al*. 2021) to evaluate genome completeness through the identification of highly conserved Ave genes from the BUSCO database. To investigate the contiguity and synteny of *D. dasypus* assembly, we employed minimap2 (Li 2018) to align its genome to the barn swallow (*Hirundo rustica*) genome [GCF_015227805.1] (Secomandi *et al*. 2023) downloaded from National Center for Biotechnology Information (NCBI) database. Subsequently, the resulting alignment was used to deduce the corresponding chromosome position for each *D. dasypus* scaffold. The final outcome was visualized using D-GENIES (Cabanettes and Klopp 2018).

### Gene annotation of *D. dasypus* genome

We performed gene annotation to the assembled *D. dasypus* genome using the BRAKER version 3.0.3 pipeline, which integrated RNA-seq data and protein homology information in gene modeling (Stanke *et al*. 2006, 2008; Iwata and Gotoh 2012; Buchfink *et al*. 2015; Hoff *et al*. 2019; Kovaka *et al*. 2019; Bruna *et al*. 2024). Specifically, we used Trimmomatic to remove adaptors and low-quality bases from *D. dasypus* RNA-seq reads (Bolger *et al*. 2014). These trimmed RNA-seq reads were subsequently mapped to the assembled *D. dasypus* genome via Hisat2 version 2.1.0 (Kim *et al*. 2019) and converted to the BAM entry format of BRAKER. For the protein homology evidence, we downloaded the Vertebrata orthologous database from OrthoDB v11 (Kuznetsov *et al*. 2023). To avoid falsely predicting gene structures in highly repetitive genomic regions, we applied RepeatMasker version 1.332 (Smit *et al*. 2013-2015) to soft-mask such genomic regions. This process was carried out with the parameter—species Passeriformes. Finally, we used the BUSCO-derived parameters to optimize the training of gene models.

### Genomic alignment

We used Progressive Cactus, a reference-free multiple genome alignment software (Armstrong *et al*. 2020), to align the genome of *D. dasypus* with those of three other swallow species—*H. rustica* [GCF_015227805.1], *Riparia riparia* [GCA_020917445.1] (Tang *et al*. 2022), and *Tachycineta bicolor* [GCA_025960845.1] (Tseng *et al*. 2023). We chose these species because they are closely related to *D. dasypus* and lack feathers on their feet (Sheldon *et al*. 2005; Supplementary Fig. 1). We downloaded their genomes from NCBI database. The soft-masked genome of *D. dasypus* was applied to reduce the potential anchors formed by the matches occurring in the region of repetitive sequences. We constructed the input guide tree with the neighbor-joining method (Saitou and Nei 1987) based on the evolutionary distance between each pair of swallow species. Specifically, we used minimap2 (Li 2018) to conduct pairwise alignment between the four swallow species. We used customized scripts in R to calculate approximate per-base sequence divergence from the alignment outputs to estimate the evolutionary distance between each pair of swallow species. Ultimately, the per-base sequence divergence was applied to construct the phylogenetic tree with the ape package in R (Paradis and Schliep 2019).

### SNP calculation and divergent window selection

To detect the most divergent regions, we analyzed single-nucleotide polymorphism (SNP) across the genomes of the four swallow species, using the genome of *D. dasypus* as a reference. In particular, we utilized the halSnps and halAlignmentDepth pipelines (Hickey *et al*. 2013) to retrieve the numbers of SNPs and aligned bases from the alignment results generated through Progressive Cactus. Subsequently, we applied customized R scripts to calculate the SNP densities per aligned base within nonoverlapping 10,000 bp windows set along the *D. dasypus* genome, based on the outputs of halSnps and halAlignmentDepth. This resulted in a set of matrices representing SNP proportions for all pairwise species comparisons.

To detect the genomic regions containing a signature of selection in *D. dasypus*, we took a comparative approach to infer high SNP proportion regions within the three focal comparisons (*D. dasypus* to *H. rustica*, *R. riparia*, and *T. bicolor*, respectively) against three control comparisons (between any two of *H. rustica*, *R. riparia*, and *T. bicolor*). To ensure the consistency of our findings, we excluded windows with the least 1% of aligned bases (3,000  bp) to mitigate potential bias stemming from SNP variations in windows with few aligned bases. Then, SNP proportions for each window were Z-transformed, expressing genome-wide differentiation in units of standard deviation relative to the mean. This step standardized all the pairwise comparisons across species. Next, we calculated delta SNP proportions (Δ*_SNP_*) by subtracting the SNP proportions in focal comparisons from the maximum value of the SNP proportion in control comparisons for each window (Vijay *et al*. 2016). Following that, we selected the top 1% windows based on Δ*_SNP_* from each of the focal comparisons. Finally, we considered the intersection windows from the three focal comparisons as prospective regions potentially related to the formation of feathered feet in the *D. dasypus* genome and called them as “selected windows”.

The comparisons above were made separately for autosomes and the Z chromosome. In birds, the Z chromosome is often subject to an elevated evolutionary rate compared to autosomes (the “fast-Z” effect), attributable to several mechanisms that render stronger selection on the Z chromosome (Xu *et al*. 2019). We also found the fast-Z effect in our focal species, revealed by greater sequence divergence between the Asian house martin and the barn swallow (the two with chromosome-level genome assemblies) on the Z chromosome than autosomes (Supplementary Fig. 2). Therefore, we conducted the above comparisons for autosomes and the Z chromosome separately. Additionally, we excluded the W chromosome from these comparisons considering that feathered feet occur in both male and female *D. dasypus*. That is, genes involved in feathered-foot formation should not reside on the W chromosome, which is exclusive to females.

In general, this approach allows the identification of *D. dasypus* genome regions driven by evolutionary processes that are specific to *D. dasypus* while avoiding the inclusion of regions governed by evolutionary processes common to different swallow species.

### Selected window composition analysis

We employed the software MULTOVL to determine whether the selected windows tended to be concentrated in the genic regions (Aszodi 2012). For this, we first calculated the overlapping length between the selected windows and the genic regions predicted by BRAKER. We then compared the resultant value against a null distribution, generated utilizing the multovlprob command within MULTOVL by reshuffling our selected windows 10,000 times across the genome. We considered the selected windows enriched or depleted in the genic regions when obtaining a positive or a negative *z* value, respectively, along with a *P*-value less than 0.05 from such a permutation test.

### Gene function analysis

We investigated whether these selected windows overlapped or were in proximity to genes predicted by BRAKER. A previous study suggested that most enhancers are less than 100 kb away from their target genes (Fulco *et al*. 2019), whereas another study indicated that enhancer–target interactions can occur over 250 kb (Vierstra *et al*. 2014). Therefore, we identified genes with start codons situated either 250 kb upstream or downstream of our selected windows as putative regulatory targets of those windows (Lu *et al*. 2023). Subsequently, we inferred their functionalities through cross-species orthologous relationships. To undertake this inquiry, we compared the *D. dasypus* peptide sequences generated by BRAKER against the sequences of two other avian species—chicken (GRCg6a) and zebra finch (bTaeGut1_v1.p), sourced from Ensembl version 103 (Martin *et al*. 2023). The latter two avian species served as a reference to establish the orthologous relationship. We then applied custom R scripts, aided by the Biostrings package, to select the longest peptide isoform of each gene (Pages *et al*. 2022) to be the entry of the orthology analysis, carried out with SONICPARANOID version 1.3.0 (Cosentino and Iwasaki 2019). The genes that were in proximity to the high SNP density windows, along with their orthologous counterparts in the chicken and zebra finch, were used for further analysis.

To assess whether the genes within 250 kb upstream or downstream of the selected windows were enriched in specific biological functions, we performed enrichment analysis using two distinct tools: the Database for Annotation, Visualization and Integrated Discovery (DAVID) Functional Annotation tool (Huang *et al*. 2009a, 2009b) and WEB-based GEne SeT AnaLysis Toolkit (WebGestalt) (Zhang *et al*. 2005; Wang *et al*. 2013, 2017; Liao *et al*. 2019). For the enrichment analysis, the selected genes, along with their corresponding Ensembl IDs in the zebra finch and chicken, were retrieved as the input gene list. In this process, the zebra finch and chicken were designated as reference species for DAVID and WebGestalt, respectively. Our analysis primarily centered on genes in terms of the Kyoto Encyclopedia of Genes and Genomes (KEGG) pathways and Gene Ontology (GO) biological process categories (Ashburner *et al*. 2000; Kanehisa and Goto 2000). We considered enrichment terms significant if their false discovery rates (FDRs) were less than 0.05.

Furthermore, we used STRING 12.0 (Szklarczyk *et al*. 2023) to investigate protein–protein interactions for genes overlapped with our selected windows. We input their protein symbols individually to detect whether our overlapped genes interacted with feathered feet linked genes. The zebra finch was selected as a reference species in this investigation. Additionally, we inspected 793 genes, whose start codons are within 250 kb upstream or downstream of our selected windows, and investigated if their functions are related to feathered feet by conducting literature searches.

### Identification of inversion regions

To explore the possibility that chromosomal inversions were associated with the feathered feet of *D. dasypus*, we identified inversion regions along with genes on them. To this end, we used minimap2 to map the *H. rustica* genome onto our *D. dasypus* genome for inversion identification as described below. We did not apply this method to either *R. riparia* or *T. bicolor* because these two species lacked chromosome-level assemblies, which prevented reliable inversion identification. On the synteny plot generated using D-GENIES, we followed Bentley *et al*. (2023) to screen 20 Mb windows for inversions of >50 kb. We then identified genes overlapped with the inversion regions and investigated their relationship with the feathered-foot trait via literature search and STRING. Additionally, SONICPARANOID version 1.3.0 was used to infer orthologs of these overlapping genes in the chickens and zebra finch, respectively. Finally, we conducted enrichment analysis using DAVID and WebGestalt as described above.

## Results and discussion

### *D. dasypus* genome quality and gene annotation

The Hi-C heatmap revealed high contiguity in the *D. dasypus* genome assembly, containing at least 31 large scaffolds, each of which presented a chromosome (Fig. 1b). The assembled *D. dasypus* genome showed highly conserved synteny with the chromosome-level assembly of *H. rustica*, demonstrated by the D-GENIES plot (Fig. 1c). The Quast results showed the *D. dasypus* genome assembly containing 321 scaffolds with a total size of 1.16 Gb and a scaffold N50 as 74,747,089 bp. The largest scaffold was 155,081,786 bp (Table 1). Among the total 8,338 BUSCO groups searched, the genome assembly demonstrated 96.8% of completeness in terms of BUSCO copies (Table 2). Remarkably, 8,021 (96.2%) of the identified BUSCOs were categorized as complete single-copy ones, indicating the successful retrieval of a majority of single-copy orthologous genes within the assembly. A minor fraction of complete BUSCOs, specifically 49 (0.6%), were identified as duplicated genes. This result potentially signifies some instances of gene duplication during the genome assembly process. Through the BRAKER pipelines, we annotated 16,165 protein-coding genes and 21,979 transcripts. Repeat regions accounted for 15.37% of the assembled genome, totaling 177,644,364 bp, as indicated by RepeatMasker (Supplementary Table 1).

**Table 1.** The de novo assembly metrics of *D. dasypus* genome.

Assembly statistics	Value
Largest scaffold (bp)	155,081,786
Total length (bp)	1,155,721,394
Total scaffolds	321
Scaffolds (>=5,000 bp)	313
Scaffolds (>=50,000 bp)	199
Scaffold N50 (bp)	74,747,089
GC (%)	42.91
*N* (%)	2.22

All statistics are from Quast based on scaffolds of size >500 bp.

**Table 2.** Summarized benchmarking in BUSCO notation for the *D. dasypus* genome based on 8,338 Ave genes.

Assembly statistics	Value
Complete genes	8,070 (96.8%)
Complete and single-copy genes	8,021 (96.2%)
Complete and duplicated genes	49 (0.6%)
Fragmented genes	75 (0.9%)
Missing genes	193 (2.3%)
Total BUSCO genes used	8,338

Percentage of each category is shown in parentheses.

### Genomic alignment and selected window identification

We conducted whole-genome alignment between *D. dasypus* and the other three swallow species with Progressive Cactus to identify the SNP proportions among these species. The percentages of the *D. dasypus* genome that aligned with other swallow species were 88.82% for *H. rustica*, 83.65% for *R. riparia*, and 87.9% for *T. bicolor*, respectively. The genome-wide proportions of SNPs against the three swallow species were 0.030 for *H. rustica*, 0.038 for *R. riparia*, and 0.039 for *T. bicolor*, as indicated by the halSnps pipeline. The computing time of the Progressive Cactus alignment for the four swallow genomes using a server with 47 available CPUs and 792,518,964 kB RAM was almost 2 weeks. Thus, the long computing time is one limitation of this approach.

**Fig. 2.**
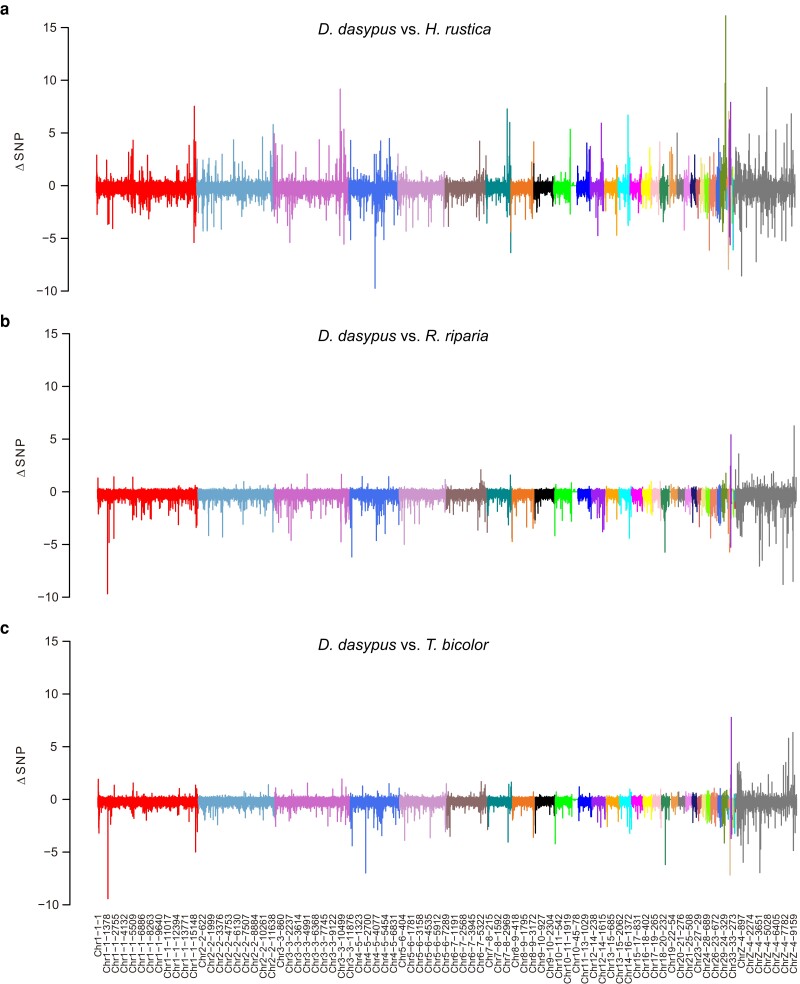
Genetic divergence (Δ*_SNP_*) between *D. dasypus* and three other swallow genomes. The Δ*_SNP_* values of 10,000 bp windows are shown across the *D. dasypus* genome. Each panel indicates the result from the alignment of the *D. dasypus* genome with three swallow species (a) *H. rustica*; (b) *R. riparia*; (c) *T. bicolor*. In each panel, distinct color regions represent a corresponding chromosome in *H. rustica*, with a total of 35 chromosomes being considered. Each label on the *x*-axis consisted of three components: ChrN1-N2-N3, where N1, N2, and N3 represent three numbers. The first component, ChrN1, designates the respective chromosome in *H. rustica* to which *D. dasypus* is aligned. The second component, N2, signifies the original scaffold number in the *D. dasypus* genome. The third component, N3, denotes the window ID within the corresponding scaffold.

**Fig. 3.**
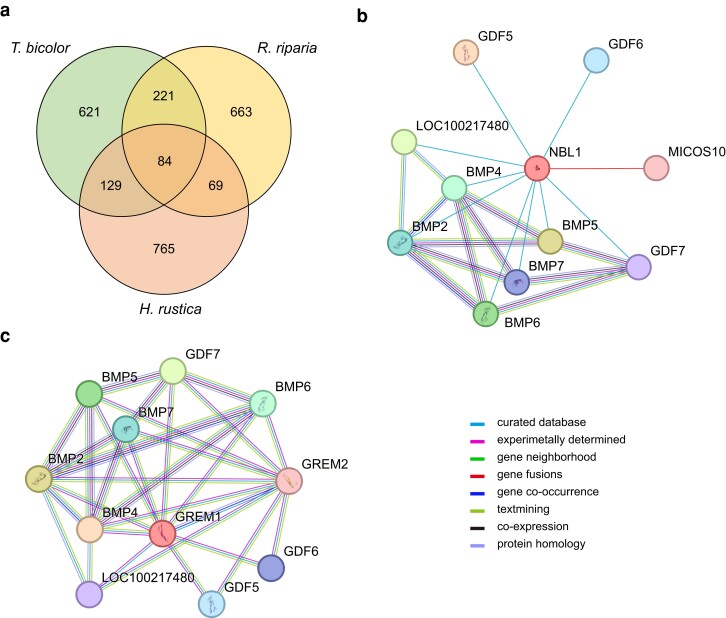
a) The Venn diagram illustrates the intersections of windows identified with top 1% Δ*_SNP_* values between *D. dasypus* and each of the other three swallow species. STRING protein–protein interactions of (b) NBL1 and (c) GREM1. The colors of lines among proteins indicate types of protein–protein association.

To detect regions with high SNP densities potentially linked to the development of feathered feet, we investigated the SNP proportions within each 10,000 bp window across the *D. dasypus* genome against the three swallow species (Fig. 2). Overall, the *D. dasypus* genome was partitioned into 115,729 windows. After removing windows with less than 3,000 aligned base pairs and the W chromosome, the average SNP proportion per window between *D. dasypus* and *H. rustica* was 0.028, whereas the average SNP proportions per window for *D. dasypus* against *R. riparia* and *T. bicolor* were 0.035 and 0.037, respectively. The window with the highest SNP proportion in autosomes was identified between *D. dasypus* and *T. bicolor*, with a value of 0.247. In the Z chromosome, the highest SNP proportion was detected between *D. dasypus* and *H. rustica*, with a value of 0.24. Instead, after calculating the Δ*_SNP_*, the minimum and maximum values, −9.73 and 16.12, both occurred in the comparison between *D. dasypus* and *H. rustica* (Fig. 2). Among the windows showing top 1% Δ*_SNP_* values, 80 windows in autosomes and four windows in the Z chromosome were concurrently identified in the alignments against all three swallow species (Fig. 3a). The Δ*_SNP_* proportions within the 84 selected windows varied from 0.208 to 7.779, and these windows were distributed around 21 chromosomes and 24 scaffolds. Among the 84 selected windows, 52 were completely located in intergenic regions, while the remaining 32 windows have 39 genes that either completely or partially overlapped with these windows (Supplementary Table 2).

### Selected window composition analysis

We employed the software MULTOVL to determine whether the 84 selected windows tended to overlap with the genic regions of the genome. Our findings exhibited a total overlap length of 217,987 bp between the genic regions and selected windows. The permutation test revealed significant depletion of the selected windows in the genic regions (*P*-value = 0.044, *z* score = −1.708). In other words, the divergent regions between *D. dasypus* and the other three swallow species were enriched in intergenic regions.

### Enrichment analysis of selected window-associated genes

We identified 793 genes with their start codons situated within the regulatory range of the 84 selected windows, spanning 250 kb both upstream and downstream (Supplementary Table 3). To explore the potential biological processes and pathways of these 793 genes, we performed enrichment analysis of their orthologous counterparts in chicken and zebra finch, respectively. Overall, the enrichment analysis of the 592 chicken and the 602 zebra finch orthologs revealed no significantly enriched GO terms in biological process and KEGG pathways. The top 10 enriched GO terms in biological processes and KEGG pathways are listed in the Supplementary Tables 4 and 5.

### Functions of selected window-associated genes

We investigated the biological functions of the 793 genes within 250 kb of the selected windows and identified six genes—Feather beta keratin-like, FGF6, FGF23, BMP10, NBL1, and WLS that may have connections with known genes associated with feathered foot development (Table 3). Our results, consistent with previous studies, suggest that several signaling pathways, such as the fibroblast growth factor (FGF), bone morphogenetic protein (BMP), and WNT pathways are involved in feather morphogenesis (Chen *et al*. 2015; Benton *et al*. 2019).

**Table 3.** Six genes potentially associated with the feathered-foot trait of *D. dasypus*.

Chr.	Scaf.	Symbol	Gene description	Source
1	1		Feather beta keratin-like	250 kb range
4	5	FGF6	Fibroblast growth factor 6	250 kb range
4	5	FGF23	Fibroblast growth factor 23	250 kb range
9	10	WLS	wntless Wnt ligand secretion mediator	250 kb range
22	26	NBL1	Neuroblastoma 1 (BMP antagonist)	250 kb range
26	23	BMP10	Bone morphogenetic protein 10	250 kb range

Scaf indicates the scaffold position of the genes and Chr indicates their corresponding chromosome in *H. rustica*. Two hundred and fifty kb range indicates the genes were identified with the 250 kb range of the selected windows.

The FGF and WNT pathways function as activators in the process of feather development (Yang *et al*. 2019; Dhouailly 2023; Du *et al*. 2023). Specifically, the activation of WNT signaling induces the expression of ectodysplasin A receptors (Edar) during embryonic development (Benton *et al*. 2019; Dhouailly 2024). Subsequently, Edar triggers FGF signals, setting the foundation for the growth of feather buds. Additionally, the FGF and WNT pathways were reported to induce feathered feet in chickens via the interaction of TBX5 and TBX4 during limb development (Takeuchi *et al*. 2003; Boer *et al*. 2019). Our study suggests a potential involvement of the three genes (WLS, FGF6, and FGF23) related to the FGF and WNT pathways in development of the feathered-foot trait in *D. dasypus*, although we could not find the involvement of TBX genes in this process. FGF6 and −23 are paralogs of FGF2, −3, −4, −10, and −20 (Stelzer *et al*. 2016). This is consistent with Yang *et al*. (2019) proposing FGF3 and FGF4 as potential candidates for the feathered-foot trait in chickens. Additionally, Chen *et al*. (2015) further fortified the importance of FGF2, −4, −10, and −20 in feather pattern formation. However, it is important to note that direct evidence supporting these associations is currently lacking.

Unlike the WNT and FGF pathways, the BMP pathway is known as an inhibitor in feather bud formation (Zou and Niswander 1996). The suppression of the BMP pathway has been shown to induce the conversion of scales to feathers in avian species (Zou and Niswander 1996). In our study, we identified two genes associated with the BMP pathway, BMP10 and NBL1. The interactions between NBL1 and BMPs were supported by the STRING analysis (Fig. 3b, Supplementary Table 6). NBL1 encodes a member of the BMP antagonist proteins, which binds to BMPs, preventing their interaction with receptors (Stelzer *et al*. 2016). The inhibition of the BMP pathway in feathered-foot traits was also exhibited in Zou and Niswander (1996), which utilized a dominant-negative type I BMP receptor to block BMP signaling, triggering the scale-to-feather transformation in chicken embryos. Moreover, a study suggests that the BMP signaling pathway may interact with TBX5 during limb development in feathered-foot pigeons (Boer *et al*. 2019). Despite different genes being implicated in feathered-foot development in chickens and Asian house martins, the similar underlying mechanism of BMP suppression suggests that the same traits can convergently evolve under distinct selection forces in different species.

### Identification of inversion regions and their role in the feathered-foot trait

Long-read sequencing has been demonstrated to be particularly powerful for identifying various genomic rearrangements, including chromosomal inversions of interest here (Chaisson *et al*. 2015; Chen *et al*. 2023). In this study, a total of 27 inversions distributed in 11 scaffolds were identified between *D. dasypus* and *H. rustica* (Fig. 1c). Baudrin *et al*. (2023) used a similar method to detect more than a dozen of inversions between the chromosome-level genomes of two Muscicapidae species, the spotted flycatcher (*Muscicapa striata*) and the collared flycatcher (*Ficedula albicollis*). In contrast, by compiling karyotypic data from 111 studies that covered 59 passerine families, Hooper and Price (2017) identified a median of only five within-family inversions (in a range of 0–83, with 1–45 species examined per family). However, the low inversion numbers were partially caused by the fact that only pericentric inversions were counted in that study (Hooper and Price 2017).

The occurrence of genome inversion has been reported to be associated with phenotypic changes (Sanchez-Donoso *et al*. 2022). To examine the link of these inversions to phenotypic traits, we investigated the genes within these regions. Overall, there were 848 genes either located within or overlapping these regions. Enrichment analysis did not exhibit any significant GO terms in biological processes or KEGG pathways among these genes. Importantly, we detected three genes—FGF3, GREM1 and BMP10—that are related to the feather development. These results imply the potential involvement of the FGF and BMP pathways in feathered-foot development.

Among these genes, two genes are related to the BMP pathway. BMP10 is the only gene that was detected simultaneously based on high-SNP and inversion regions. Additionally, similar to NBL1 identified earlier, GREM1 acts as a BMP antagonist in the BMP pathway and plays a role in scale-to-feather conversion (Hughes *et al*. 2011; Wu *et al*. 2018). Previous studies indicated GREM1 regulate the BMP signaling to modulate feather topology (Li *et al*. 2017; Ng and Li 2018; Dhouailly 2024). Its involvement in feather formation is further supported by our protein–protein interactions exhibited by the STRING results (Fig. 3c, Supplementary Table 6). Both GREM1 and NBL1 downregulate the two proteins, BMP2 and BMP7, which are shown to be responsible for feather induction in chickens (Michon *et al*. 2008). Moreover, consistent with Yang *et al*. (2019), we found FGF3 potentially linked to the feathered-foot trait. Chen *et al*. (2015) also indicates that its paralogs, FGF2, −4, −10, and −20 could play roles in feather development. Overall, the genes identified in the chromosomal inversions and high-SNP regions highlight the differentiation of feather-related traits among swallow species, with a potential link to foot feathering.

However, the potential genes linked to feathered feet identified by the chromosomal inversions are less convincing compared to high-SNP regions since we only aligned the genomes of *D. dasypus* and *H. rustica* for this analysis. Therefore, any structure variant revealed by this analysis only reflected the difference in evolutionary paths between *D. dasypus* and *H. rustica*. Feathered feet are one of the multiple traits in *D. dasypus* to be implied in the inversion regions. This limitation stems from the lack of chromosome-level assemblies for *R. riparia* and *T. bicolor*, hampering our investigation of structure variants between *D. dasypus* and other swallows. Future studies are recommended to assemble chromosome-level genomes of other swallow species to explore structural variation underlying the formation of feathered feet in avian species.

## Conclusion

With the advancement of bioinformatic tools and sequencing technologies, whole-genome analysis has allowed researchers to comprehensively explore genetic variation, genomic architecture, and their associated pathways underlying a complex trait (Almasy 2012; Bocher *et al*. 2023). Here, we presented a chromosome-level genome of the Asian house martin. The whole-genome analysis on this and other three swallow species hints at genes related to its feathered-foot trait. These genes are parts of the WNT, BMP, and FGF pathways, which have been previously linked to limb development and feather morphogenesis, including feathered foot development. Although these genes are not the same as those identified in previous studies on domestic populations, our findings still imply the presence of similar biological pathways regulating the genetic mechanisms of ptilopody. That is, the convergent evolution of feathered feet in both wild and domestic birds is governed by mutations in similar pathways, albeit involving different genes (Bortoluzzi *et al*. 2020).

Foot feathering is a trait observed in certain orders among modern birds, such as Accipitriformes, Strigiformes, and Pterocliformes (Billerman 2022) and in extinct bird and nonavian dinosaur lineages, such as *Archaeopteryx* and *Microraptor*. Its function in living raptors and extinct *Microraptor* has been proposed to be associated with aerodynamic adaptation (Chatterjee and Templin 2007). Noteworthy. *Delichon* is the only genus with foot feathering in Passeriformes (Billerman 2022). Despite this trait has evolved independently across different species, whether its function and selection pressure remain the same in *D. dasypus* is still yet to be answered. Notably, this is the first study utilizing whole-genome analysis to explore associated genes underpinning the feathered feet in a nondomestic species. Future studies are recommended to utilize the chromosome-level assembly of *D. dasypus* to conduct comparative genomic studies with other feathered-foot species, such as the golden eagle and northern hawk owl that might shed light on the evolutionary processes shaping ptilopody. This will potentially bridge the evolutionary gap in feathered feet between modern birds and nonavian dinosaurs, offering valuable insights into the origin of feathers in avian species.

## Supplementary Material

jkae077_Supplementary_Data

## Data Availability

*Delichon dasypus* genomic sequencing (PacBio HiFi and Hi-C) and RNA-seq reads are deposited in the NCBI Sequence Read Archive under the accession number PRJNA1049492. The GenBank accession for the assembled genome is JBBAXF000000000. The gene annotation and codes used in this study are available in the FigShare repository (doi:10.6084/m9.figshare.25215875). Supplemental material available at G3 online.
