# Supplementary material for: A chromosome-level genome assembly of the Asian house martin implies potential genes associated with the feathered-foot trait
Source: G3 (Bethesda). 2024 Apr 12;14(6):jkae077. doi: 10.1093/g3journal/jkae077 (PMC11152083; doi:10.1093/g3journal/jkae077)
Supplement: jkae077_Supplementary_Data [file jkae077_supplementary_data.zip › Supplementary_Figure_1_G3-2024-404966.docx]

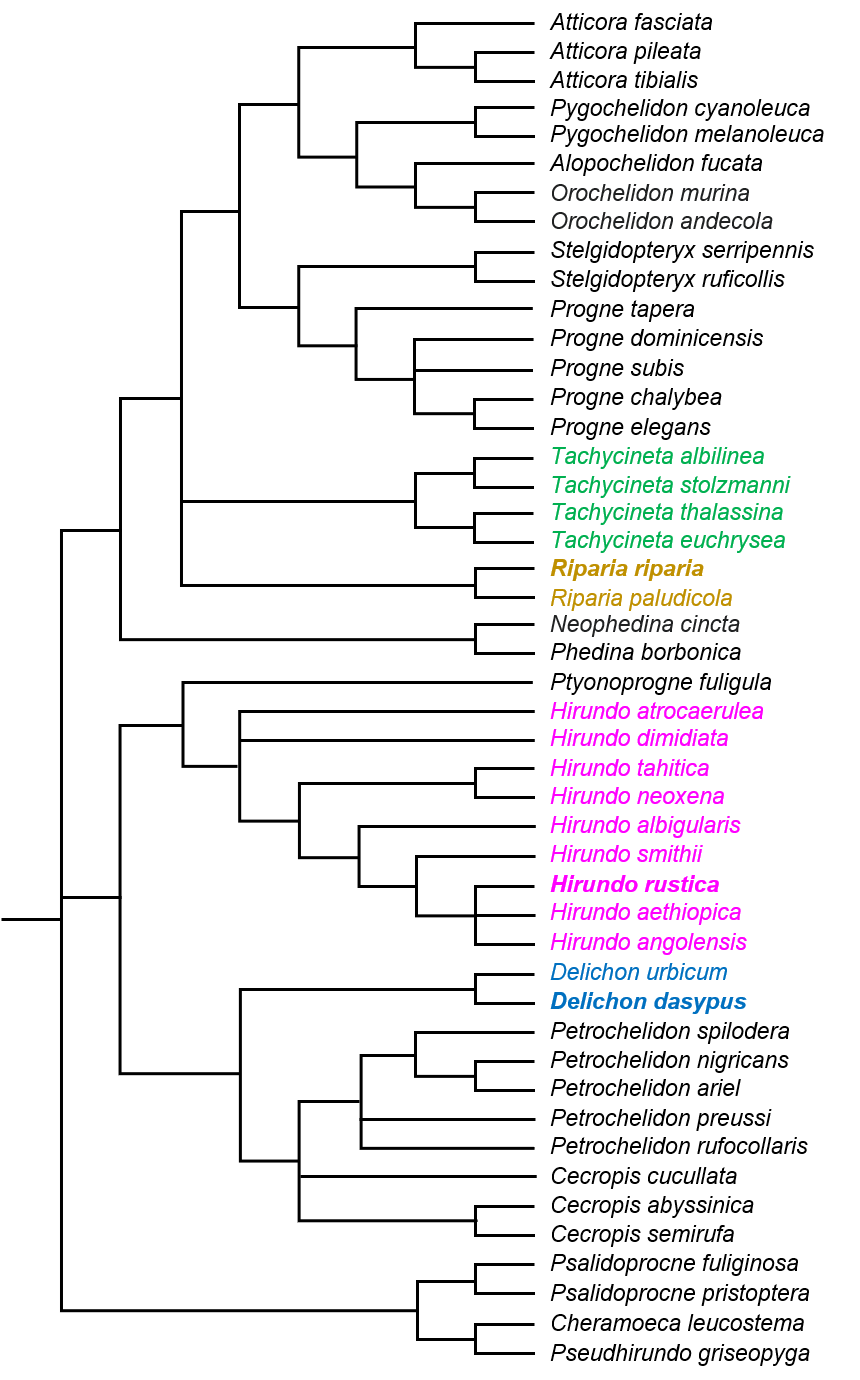


**Supplementary Figure 1.** A Bayesian phylogenetic tree for 47 Hirundininae species, showing only the topology. The tree is modified after Sheldon et al. (2005) who reconstructed it based on two mitochondrial (cytochrome *b* and ND2) and one nuclear (β-fibrinogen intron 7) markers. Inferred relationships with < 0.95 posterior probability support are collapsed. The four swallow species currently studied have their belonging genera differently colored, and the species themselves, if present, are shown in bold.
