# Supplementary material for: A chromosome-level genome assembly of the Asian house martin implies potential genes associated with the feathered-foot trait
Source: G3 (Bethesda). 2024 Apr 12;14(6):jkae077. doi: 10.1093/g3journal/jkae077 (PMC11152083; doi:10.1093/g3journal/jkae077)
Supplement: jkae077_Supplementary_Data [file jkae077_supplementary_data.zip › Supplementary_Figure_2_G3-2024-404966.docx]

(a)

(b)

**Supplementary Figure 2.** Autosome (A) vs. Z chromosome (Z) divergence levels between the Asian house martin and the barn swallow. (a) The levels of sequence divergence against the alignment length. The alignment is conducted by minimap2. Based on the plot, we choose alignments of ≥10,000 bp (when the divergence levels converged to the chromosome-wide levels) for the A-Z comparison. (b) It shows significantly greater divergence on the Z chromosome than on the autosomes. The Mann-Whitney test is used to evaluate the A-Z difference, with *** indicating P <0.001.
