# Supplementary material for: A chromosome-level genome assembly of the Asian house martin implies potential genes associated with the feathered-foot trait
Source: G3 (Bethesda). 2024 Apr 12;14(6):jkae077. doi: 10.1093/g3journal/jkae077 (PMC11152083; doi:10.1093/g3journal/jkae077)
Supplement: jkae077_Supplementary_Data [file jkae077_supplementary_data.zip › Supplementary_Table_1_G3-2024-404966.docx]

**Supplementary Table 1.** Repetitive elements identified in the *D. dasypus* genome.

| Classification | Number of elements | Length occupied | Percentage of sequence |
| --- | --- | --- | --- |
| SINEs | 871 | 73303 bp | 0.01 % |
| LINEs | 124705 | 38266295 bp | 3.31 % |
| LTR elements | 101844 | 82343086 bp | 7.12 % |
| DNA elements | 7562 | 1505416 bp | 0.13 % |
| Unclassified | 34439 | 32624038 bp | 2.82 % |
| Satellites | 4857 | 3289936 bp | 0.28 % |
| Simple repeats | 315485 | 16264134 bp | 1.41 % |
| Low complexity | 55860 | 3292760 bp | 0.28 % |

SINE, short interspersed nuclear element; LINE, long interspersed nuclear element; LTR, long terminal repeat
