# Supplementary material for: A chromosome-level genome assembly of the Asian house martin implies potential genes associated with the feathered-foot trait
Source: G3 (Bethesda). 2024 Apr 12;14(6):jkae077. doi: 10.1093/g3journal/jkae077 (PMC11152083; doi:10.1093/g3journal/jkae077)
Supplement: jkae077_Supplementary_Data [file jkae077_supplementary_data.zip › Supplementary_Table_4_G3-2024-404966.docx]

**Supplementary Table 4.** The top 10 KEGG enrichment results from 793 genes around the selected windows.

| Gene Set | Description | Size | P Value | FDR |
| --- | --- | --- | --- | --- |
| gga04144 | Endocytosis | 224 | 0.014802 | 1 |
| gga00910 | Nitrogen metabolism | 17 | 0.021766 | 1 |
| gga00592 | alpha-Linolenic acid metabolism | 25 | 0.059705 | 1 |
| gga04210 | Apoptosis | 122 | 0.073247 | 1 |
| gga04330 | Notch signaling pathway | 44 | 0.073179 | 1 |
| gga00591 | Linoleic acid metabolism | 27 | 0.072092 | 1 |
| gga00604 | Glycosphingolipid biosynthesis | 13 | 0.078133 | 1 |
| gga03040 | Spliceosome | 107 | 0.038873 | 1 |
| gga00565 | Ether lipid metabolism | 44 | 0.073179 | 1 |
| gga00051 | Fructose and mannose metabolism | 34 | 0.032914 | 1 |
