# Supplementary material for: A chromosome-level genome assembly of the Asian house martin implies potential genes associated with the feathered-foot trait
Source: G3 (Bethesda). 2024 Apr 12;14(6):jkae077. doi: 10.1093/g3journal/jkae077 (PMC11152083; doi:10.1093/g3journal/jkae077)
Supplement: jkae077_Supplementary_Data [file jkae077_supplementary_data.zip › Supplementary_Table_5_G3-2024-404966.docx]

**Supplementary Table 5.** The top 10 Go enrichment results from 793 genes around the selected windows.

| Gene Set | Description | Size | P Value | FDR |
| --- | --- | --- | --- | --- |
| GO:0140053 | mitochondrial gene expression | 44 | 0.0060253 | 1 |
| GO:0006383 | transcription by RNA polymerase III | 23 | 0.010281 | 1 |
| GO:0043603 | cellular amide metabolic process | 427 | 0.020201 | 1 |
| GO:0009607 | response to biotic stimulus | 262 | 0.021781 | 1 |
| GO:0006886 | intracellular protein transport | 388 | 0.022347 | 1 |
| GO:0007034 | vacuolar transport | 67 | 0.041440 | 1 |
| GO:0104004 | cellular response to environmental stimulus | 103 | 0.042090 | 1 |
| GO:0071806 | protein transmembrane transport | 36 | 0.046620 | 1 |
| GO:0006955 | immune response | 374 | 0.046799 | 1 |
| GO:0016458 | gene silencing | 71 | 0.052546 | 1 |
