# Supplementary material for: A chromosome-level genome assembly of the Asian house martin implies potential genes associated with the feathered-foot trait
Source: G3 (Bethesda). 2024 Apr 12;14(6):jkae077. doi: 10.1093/g3journal/jkae077 (PMC11152083; doi:10.1093/g3journal/jkae077)
Supplement: jkae077_Supplementary_Data [file jkae077_supplementary_data.zip › Supplementary_Table_6_G3-2024-404966.docx]

**Supplementary Table 6.** The confidence scores of the NBL1 and GREM1 and their interacted proteins.

| Subject | Targeted protein | Confidence score |
| --- | --- | --- |
| NBL1 | BMP2 | 0.9 |
| NBL1 | BMP4 | 0.9 |
| NBL1 | BMP5 | 0.9 |
| NBL1 | BMP6 | 0.9 |
| NBL1 | BMP7 | 0.9 |
| NBL1 | GDF5 | 0.9 |
| NBL1 | GDF6 | 0.9 |
| NBL1 | GDF7 | 0.9 |
| NBL1 | LOC100217480 | 0.9 |
| NBL1 | MICOS10 | 0.515 |
| GREM1 | BMP2 | 0.925 |
| GREM1 | BMP4 | 0.925 |
| GREM1 | BMP5 | 0.908 |
| GREM1 | BMP6 | 0.908 |
| GREM1 | BMP7 | 0.908 |
| GREM1 | GDF5 | 0.904 |
| GREM1 | GDF6 | 0.904 |
| GREM1 | GDF7 | 0.921 |
| GREM1 | GREM2 | 0.902 |
| GREM1 | LOC100217480 | 0.903 |
